# Comparison of Outcomes of Resident-performed Ahmed Valve Implantation *vs* Trabeculectomy

**DOI:** 10.5005/jp-journals-10008-1203

**Published:** 2016-08-05

**Authors:** Robert A Sharpe, Leah L Kammerdiener, Kendall W Wannamaker, Jie Fan, Elizabeth D Sharpe

**Affiliations:** 1 Resident, Department of Ophthalmology, Medical University of South Carolina, Charleston, South Carolina, USA; 2 Fellow, Department of Ophthalmology, Medical University of South Carolina, Charleston, South Carolina, USA; 3 Resident, Department of Ophthalmology, Medical University of South Carolina, Charleston, South Carolina, USA; 4 Research Instructor, Department of Ophthalmology, Medical University of South Carolina, Charleston, South Carolina, USA; 5 Professor, Department of Ophthalmology, Ralph H. Johnson Veterans Affairs Medical Center, Charleston, South Carolina, USA

**Keywords:** Glaucoma, Resident, Trabeculectomy, Training, Valve.

## Abstract

**Aims:** To compare outcomes of resident-performed Ahmed valve surgery *vs* trabeculectomy in a Veteran Affairs medical facility.

**Materials and methods:** A retrospective cohort of 103 eyes in 91 patients receiving Ahmed valve (valve) or trabeculectomy (trab) performed at a Veterans Administration Medical Center by residents in their third year of training. The primary outcomes included intraocular pressure (IOP), treatment failure, and complications over 1 year.

**Results:** Of 103 eyes, 44 received valve and 59 received trab. Primary open-angle glaucoma was primary diagnosis more often in trab, while neovascular glaucoma predominated in the valve group (p < 0.001). Preoperative mean IOP was 35.1 ± 11.8 and 24.5 ± 7.1 mm Hg for valve and trabeculectomy respectively (p < 0.001), but at 1 year the IOP difference between groups was not statistically significant (p = 0.064). Overall, 11 (25.0%) and 11 (18.6%) eyes met any criteria for failure for valve and trab respectively. At 1 year, 22.5% of valves had IOP > 21 mm Hg *vs* only 4.3% of trab (p = 0.02). Complications were infrequent. There were no intraoperative complications for valve, whereas five for trab. Most common immediate complication for valve was hyphema. Both groups had low rates of choroidal effusions and reoperation.

**Conclusion:** Ahmed valve implantation and trabeculectomy produce significant reductions in IOP when performed by residents-in-training. Valves tend to be used more frequently in patients with secondary glaucoma. Although complication profiles differ between procedures, both are safe and well tolerated when performed by resident physicians.

**Clinical significance:** This study provides support for evidence-based patient counseling that supervised, resident-performed Ahmed valve implantation and trabeculectomy are indeed safe and effective.

**How to cite this article:** Sharpe RA, Kammerdiener LL, Wannamaker KW, Fan J, Sharpe ED. Comparison of Outcomes of Resident-performed Ahmed Valve Implantation *vs* Trabeculectomy. J Curr Glaucoma Pract 2016;10(2):60-67.

## INTRODUCTION

Glaucoma is a progressive optic neuropathy, i.e., a leading cause of irreversible blindness worldwide.^1^ The mainstay of treatment centers on reducing intraocular pressure (IOP). Surgical procedures are commonly employed in the management of glaucoma when medical and laser treatments are inadequate or not tolerated.^2^

Trabeculectomy, first described in 1968, remains the gold standard and most commonly performed incisional glaucoma surgery.^3-5^ Trabeculectomy is typically performed with antifibrotic agents, most commonly mito-mycin C, to improve the success rates of bleb function.^6^ Despite its widespread acceptance, trabeculectomy with antifibrotic agents is a technically demanding procedure and requires careful follow-up to produce optimal aqueous outflow without overfiltration.

The Ahmed valve (New World Medical, Rancho Cucamonga, California) is a tube shunt that has traditionally been reserved for select patients with an increased risk of trabeculectomy failure or a high risk of secondary glaucoma.^7,8^ More recently, tube shunts have grown in popularity as the first-line surgery for glaucoma.^3,4,6,9^ The advantage of the Ahmed valve, like all tube shunts, lies in its relatively straightforward implantation process and has been shown to require fewer postoperative interventions than the nonvalved Baerveldt at 1 year.^10^

During their 3 years of training, ophthalmology residents are required to perform various surgical treatments for glaucoma. The American College of Graduate Medical Education requires residents to log at least five filtering or shunt surgeries as primary surgeon before graduation.^11^ Eighty-four percent of residents receive surgical instruction by fellowship-trained glaucoma specialist and act as primary surgeon for a tube shunt by their third year.^12^ On average, residents perform 3.6 tube shunts and 8.6 trabeculectomies during training.^12^ Adequate exposure is essential during residency because comprehensive ophthalmologists, who generally do not complete fellowships, commonly perform these procedures when medically necessary.^13^

This study aims to investigate Ahmed valve implantation outcomes compared with trabeculectomy when performed by residents-in-training. Connor et al^14^ published a similar study in 2010, but compared trabeculec-tomy with Baerveldt devices. To our knowledge, no other study has investigated IOP outcomes and complication rates of these two glaucoma surgeries performed by residents to date.

## MATERIALS AND METHODS

A retrospective case review was performed at the Ralph H Johnson Veterans Affairs Medical Center (VAMC) in Charleston, SC. The study was approved by the Medical University of South Carolina Institutional Review Board and the VAMC Office of Research and Development. All cases were performed by residents in their third year of ophthalmology training. All stages of this study were conducted in accordance with the principles set forth by the Declaration of Helsinki.

Patients who underwent glaucoma surgery from 2005 to 2012 were identified via surgery schedules. All included surgeries were performed under the supervision of a single, fellowship-trained glaucoma surgeon. From that list, only primary Ahmed valve implantation and trabeculectomy cases were included. Preoperative clinic visits within 6 months of the surgical dates were used for baseline parameters. Patients were then followed as a retrospective cohort for 1 year postoperatively.

Patients with prior incisional glaucoma surgery were excluded from this study. Additional exclusion criteria consisted of loss to follow-up before 1 month postoperatively, revision procedures, and combination procedures including cyclocryotherapy, cyclophotoco-agulation, limbal relaxing incisions, intravitreal injections, and vitrectomy. In addition, combination Ahmed valve-phacoemulsification and phacoemulsification-trabeculectomy were excluded.

Demographic data including age, sex, race, history of diabetes, and history of hypertension were collected. Ocular history was also obtained, including glaucoma diagnosis, prior nonglaucoma ophthalmic surgery, and prior glaucoma laser treatment. Intraocular pressure, number of ocular antihypertensive medications, best-corrected visual acuity (BVCA), and cup-to-disk ratio (C/D) were evaluated preoperatively and at 1 year post-operatively. Preoperative IOP was calculated by taking the mean IOP from 2 visits within 6 months of surgery. Intraocular pressure was also measured at 1 day, 1 week, 1, 3, 6 months, and 1 year. Intra- and postoperative complications were recorded over 1 year. Complications over 1 year were recorded for each type of surgery. Time course of complications was defined as immediate, early, or late for those occurring at < 1 week, 1 week to 3 months, and 3 to 6 months respectively. Overfiltration (i.e., hypotony) was defined as were defined as IOP < 5 mm Hg.

After surgery, patients returned at standard intervals for routine postoperative care unless a complication arose that necessitated closer follow-up. Patients in all groups received topical steroids (prednisolone acetate 1%) and antibiotics (most commonly moxifloxacin 0.5%) postoperatively. At each follow-up appointment, BCVA was obtained by refraction or pinhole, IOP was measured with Goldmann applanation tonometry, and slit-lamp biomicroscopy was performed. Indirect ophthalmoscopy was performed to evaluate for choroidal effusion when the IOP was < 5 mm Hg. Suture lysis was performed as needed with Argon laser between 1 and 3 weeks after trabeculectomy to increase outflow using clinical judgment based on IOP and health of optic nerve.

Criteria for failure were modeled after the Tube *vs* Trabeculectomy study.^15^ Treatment failure included IOP > 21 mm Hg, IOP not reduced by 20% below baseline on two consecutive follow-up visits after 3 months postoperatively, or IOP ≤ 5 mm Hg on two consecutive follow-up visits after 3 months. If data from two consecutive visits after 3 months were not available, then the eye was excluded from treatment failure analysis.

Ahmed valves were implanted superotemporally using standard surgical technique. A peribulbar block was administered using 5 cc of a 50% mixture of 2% lidocaine and 0.75% bupivacaine. A 6-0 plain gut bridle suture was used to rotate the eye inferonasally. Conjunctiva and Tenon’s capsule were then bluntly dissected and undermined anteriorly and posteriorly. The Ahmed valve was tested for functionality and then placed into the wound and positioned posteriorly. It was sutured in place approximately 7 mm posterior to the limbus, and the tube was cut and beveled to fit 2 to 3 mm into the anterior chamber (AC). A 23-gauge needle was used to create a tract starting 0.5 mm from the limbus into the AC, running parallel to the plane of the iris, and Healon was injected into the AC. The tube was then placed in the tract and positioned in the AC. A 10-0 nylon suture was used to secure the tube to the sclera. A Tutoplast graft patch (IOP Ophthalmics, Costa Mesa, California) was then sutured with 10-0 nylon over the valve and tube. Tenon’s capsule and conjunctiva were reapproximated and closed, and neomycin/polymyxin B/dexamethasone ophthalmic ointment was applied at the end of the case.

A superotemporal or superonasal approach was employed for all trabeculectomies. Peribulbar block with 5 cc of a 50% mixture of 1% lidocaine and 0.75% bupivic-aine was performed. The eye was rotated inferonasally with a bridle suture. Careful blunt dissection was used to take free conjunctiva and separate it from Tenon’s capsule. A 50% partial thickness, rectangular scleral flap was created with a sclerotome. Mitomycin C (MMC) (0.2 mg/mL) was placed onto the sclera with a Weck-Cel sponge (Beaver Visitec, Waltham, Massachussetts) for 5 minutes followed by copious irrigation. After a para-centesis, a clear corneal incision was made underneath the scleral flap with a 15° slit blade. A Kelley punch was used to create the trabeculectomy. A surgical iridec-tomy was performed. The scleral flap was then sutured into place usually with two 10-0 nylon sutures at the corners of the flap, allowing for a small flow of aqueous through the posterior edge of the flap. Careful, watertight closure of the conjunctiva was accomplished with a 9-0 vicryl suture. At the end of each case, topical neomycin/ polymyxin B/dexamethasone ophthalmic and atropine ointment were applied.

Residents were permitted to perform glaucoma surgery as primary surgeons only after the supervising attending felt confident in a resident’s surgical skills, usually via performance in phacoemulsification. Because all procedures were supervised by the same attending supervisor, the surgical techniques and postoperative management employed were consistent throughout the study.

Data were recorded and analyzed using MS Excel (Microsoft, Redmond, WA). For statistical analysis, simple descriptive statistics, such as mean, range, and standard deviation were calculated for all outcome variables. Snellen BCVA was converted to logMAR for statistical analysis. For comparisons of categorical variables, the chi-square test or Fischer’s exact test was performed, while the Student’s t-test, Mann-Whitney U test, or Analysis of Variance (ANOVA) was performed for continuous variables. All analyses were two-way, and significance was defined as p-value of 0.05 or less.

## RESULTS

In total, 272 glaucoma surgeries were performed by third-year ophthalmology residents between 2005 and 2012. A total of 103 eyes of 91 patients were included, with 44 receiving an Ahmed valve and 59 receiving a trabeculectomy (Table 1). Patients were of mean age of 68.1 ± 12.3 and 61.6 ± 9.1 years for valve and trab respectively (p = 0.005). In both the groups, majority of the patients were male and African American (p ≥ 0.25). Majority of the patients also carried a diagnosis of hypertension as documented in their medical record. Proportionally, more patients in the valve group carried a diagnosis of diabetes mellitus (p = 0.002); however, the mean glycosyl-ated hemoglobin A1c values within 6 months of surgery were not statistically significant between groups (p = 0.10).

Specific glaucoma diagnoses in each group are summarized in Table 2. Eyes receiving a valve more commonly carried a diagnosis of neovascular glaucoma at 41 *vs* 0% for trabeculectomy (p < 0.001). For the trabeculectomy group, 97% carried a diagnosis of primary open-angle glaucoma (POAG) *vs* 36% for valves (p < 0.001). Other diagnoses, including uveitic glaucoma and chronic angle closure, were not statistically different between groups (p > 0.07).

Preoperatively, mean IOP was higher in eyes receiving a valve than trabeculectomy at 35.1 ± 11.8 and 24.5 ± 7.1 mm Hg for valve and trabeculectomy respectively (p < 0.001) (Table 3). Preoperative logMAR visual acuity was higher, indicating worse visual acuity, for eyes receiving a valve by 0.775 (p < 0.001); however, the mean C/D ratio was smaller in the valve group by 0.15 (p = 0.002). The number of ocular antihypertensive medications did not differ statistically between groups (p = 0.48).

**Graph 1:**
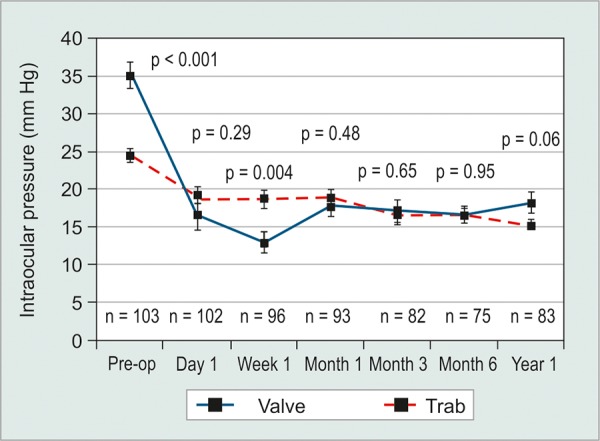
Mean intraocular pressure (± SEM) curve over 1 year for Ahmed valve (solid line) and trabeculectomy (dashed line). N = number of eyes measured at a particular time point

At 1 year postoperatively, mean IOP for valve and trabeculectomy were 18.2 ± 8.3 and 15.3 ± 4.5 mm Hg respectively (p = 0.064) (Table 3). Neither the mean number of ocular antihypertensive medications or C/D ratio was statistically different between groups at 1 year (p > 0.12). However, the mean logMAR visual acuity remained significantly poorer in the valve group, measuring 1.35 ± 1.16 and 0.57 ± 0.69 for valve and trabeculectomy respectively (p = 0.001). When comparing preoperative to 1 year postoperative IOP, both surgeries demonstrated statistically significant reductions in IOP, although the magnitude was greater for valve than trabeculectomy at 16.9 ± 14.7 and 9.2 ± 7.5 mm Hg respectively (p < 0.001) (Table 4). The IOP curve over 1 year for each group is illustrated in Graph 1.

**Table 1:** **Table 1:** Demographic characteristics of study participants

				*Valve*		*Trab*		*p-value*	
				(n = 44)		(n = 59)			
Age, mean				68.1 ± 12.3		61.6 ± 9.2		< 0.01	
(SD), y									
Sex		Male		43		56		0.64	
		Female		1		3			
Race		African-American		30		47		0.25	
		Caucasian		14		12		0.25	
		Hispanic		0		0			
		Other		0		0			
Hypertension		No. (%)		39 (89)		46 (78)		0.20	
Diabetes		No. (%)		29 (66)		22 (37)		< 0.01	
		A1c %		7.98		7.17		0.10	

Valve: Ahmed valve; Trab: Trabeculectomy

**Table 2:** **Table 2:** Glaucoma diagnosis of eyes receiving valve and trab

*Diagnosis*		*Valve*		*Trab*		*p-value*	
POAG		16		57		< 0.001	
Neovascular		18		0		< 0.001	
Uveitic glaucoma		3		0		0.08	
Chronic angle closure		1		0		0.43	
Other (OHT, Pseudoexfoliation)		6		2		0.07	

**Table 3:** **Table 3:** Preoperative and 1 year postoperative metrics for Ahmed valve and trabeculectomy

				*Valve*		*Trab*		*p-value*	
*Pre-op*		IOP		35.1 ± 11.8		24.5 ± 7.1		< 0.001	
		No. of meds		3.2±1.3		3.4 ± 0.9		0.48	
		Visual acuity		1.363 ± 0.99		0.588 ± 0.742		< 0.001	
		C/D ratio		0.72 ± 0.26		0.87 ± 0.10		0.002	
*Post-op*		IOP		18.2 ± 8.3		15.3 ± 4.5		0.064	
		No. of meds		2.3 ± 1.2		2.2 ± 1.5		0.944	
		Visual acuity		1.35 ± 1.16		0.57 ± 0.69		0.001	
		C/D ratio		0.81 ± 0.20		0.88 ± 0.09		0.12	

IOP: Intraocular pressure (mm Hg). Visual acuity reported as logMAR

Regarding rates of treatment failure, 22.5% of eyes with valve had IOP > 21 mm Hg on two occasions after 3 months postoperatively *vs* only 4.3% of eyes with trabeculectomy (p = 0.02) (Table 5). Alternatively, 15.0 *vs* 21.3% exhibited < 20% IOP reduction for valve and trab-eculectomy respectively, although the difference did not reach statistical significance (p = 0.28). Neither group had eyes with IOP < 5 mm Hg on two occasions after 3 months postoperatively. Overall, 11 (25.0%) valves and 11 (18.6%) trabeculectomies met any of the three criteria for failure.

Complications from either type of surgery were overall infrequent. However, complication profiles differed for each type of surgery, as shown in Table 6. There were no intraoperative complications for valves. Four trabeculectomies were complicated by formation of buttonholes upon conjunctiva closure. The most common immediate complication for a valve implantation was hyphema (55%); while for trabeculectomy, overfiltration (33%) and hyphema (33%) predominated. Within 1 week, one eye receiving trabeculectomy developed a choroidal effusion *vs* none receiving valve. However, from 1 week to 3 months, five eyes receiving valve developed a choroi-dal effusion *vs* only one receiving trabeculectomy. Over 1 year, five eyes that received trabeculectomy developed a cataract *vs* only one that received valve.

Regarding the need to return to the operating room, three eyes that received a valve underwent reoperation within the first 3 months *vs* none who underwent trab-eculectomy. After 3 months, one eye with a valve and two with trabeculectomy required operative intervention.

**Table 4:** **Table 4:** Changes in metrics from preoperative baseline to 1 year postoperatively

		*Valve*		*p-value*		*Trab*		*p-value*	
IOP		–16.9 ± 14.7		< 0.001		–9.2 ± 7.5		< 0.001	
No. of meds		–1.0±1.5		0.001		–1.2±1.6		< 0.001	
Visual acuity		– 0.01 ± 0.68		0.34		–0.02 ± 0.28		0.81	
C/D ratio		0.09 ± 0.23		0.19		0.01 ± 0.06		0.20	

**Table 5:** **Table 5:** Rates of treatment failure for valve and trabeculectomy for patients evaluated on two occasions after 3 months postoperatively

		*Valve*						*Trab*					
		*IOP > 21 mm Hg*		*< 20% IOP reduction*		*IOP < 5 mm Hg*		*IOP> 21 mm Hg*		*< 20% IOP reduction*		*IOP < 5 mm Hg*	
No. of eyes (n)		9		6		0		2		10		0	
Total included (n)		40		40		40		47		47		47	
No. excluded (n)		4		4		4		12		12		12	
Percent failure (%)		22.5		15.0		0		4.3		21.3		0	

## DISCUSSION

In this study, Ahmed valve implantation and trabecu-lectomy were shown to be both safe and effective when performed by residents-in-training under the supervision of a glaucoma specialist at a Veterans Affairs Medical Center. While multiple other studies have compared outcomes of Ahmed valves and trabeculectomy,^9,16,17^ this is the first study comparing not only IOP changes but also complication rates of these two glaucoma surgeries when performed by residents.

Both surgical groups in this study had significant reductions in IOP over 1 year. The magnitude of reduction was greater for valves, but this was likely due to the higher mean preoperative IOP in valve group. By 12 months, there was statistical difference in IOP between valve and trabeculectomy groups. In a prospective study of 117 patients comparing Ahmed valve with tra-beculectomy when performed by glaucoma specialists, trabeculectomy maintained lower IOP than valves, with the difference being statistically significant by 1 year.^9^ However, when that same cohort was evaluated in a long-term follow-up study, there was no difference found in IOP between groups by months 41 to 52.^16^ An important difference between their cohort and the present study is the baseline characteristics of the participants, who were on average approximately 10 years younger and more evenly divided by sex. Glaucoma diagnosis was also more matched between surgical groups, and the trab group had lower mean preoperative IOP, making these eyes arguably more favorable in terms of prognosis.

**Table 6:** **Table 6:** Postoperative complications for each group over 1 year recorded as n(%). Percentages relate to total number of complications for a particular surgery in the given timeframe

		*Immediate (< 1 week)*				*Early (1 week-3 months)*				*Late (3-12 months)*			
		*Valve*		*Trab*		*Valve*		*Trab*		*Valve*		*Trab*	
Overfiltration/hypotony				3 (33)		1 (5)		2 (14)		1 (14)		1 (8)	
Leak		1 (5)		1 (11)				1 (7)					
Tube occlusion		3 (15)				1 (5)							
Flat anterior chamber						1 (5)				1 (14)			
Corneal complications						1 (5)							
Hyphema		11 (55)		3 (33)		1 (5)		2 (14)					
Cataract		1 (5)				1 (5)		1 (7)				4 (33)	
Synechiae						1 (5)							
Blebitis													
Tube malposition		1 (5)				3 (16)				1 (14)			
Tube erosion													
Choroidal effusion				1 (11)		5 (26)		1 (7)		1 (14)			
Suprachoroidal hemorrhage				1 (11)				1 (7)					
Endophthalmitis													
Vision decrease/loss of light perception		1 (5)				1 (5)		2 (14)				2 (17)	
Reoperation		2 (10)				1 (5)				1 (14)		2 (17)	
Dry eye from bleb/valve		1 (5)						2 (14)		1 (14)		2 (17)	
Excessive inflammation						1 (5)		2 (14)		1 (14)		1 (8)	
Vein occlusion													
Motility disorder													
Diplopia													
Vitreous hemorrhage													
Central retinal vein occlusion													
Epiretinal membrane													
Retinal/choroidal detachment						1 (5)							
Cystoid macular edema													
Hypotony maculopathy													
Total (n)		21		9		18		14		7		12	

Based on criteria used in the TVT trial for treatment failure,^15^ the valve group had more failures due to persistent IOP > 21 mm Hg than trabeculectomy, while more in the trab group producing a less than 20% reduction in IOP. Overall failure rates were higher in valve group. Again, this is likely a result of higher mean IOP in the valve group and also more severe disease processes on average in that group. The fewer eyes with IOP reduction by at least 20% in the trabeculectomy group may be due to the lower preoperative IOP in that group. No eyes were classified as treatment failures based on having IOP < 5 mm Hg on two consecutive visits. In the TVT study, Gedde et al^15^ reported 4 and 13% rates for treatment failure for tube *vs* trabeculectomy respectively. Not only did the TVT include Baerveldt tube shunt instead of Ahmed valve, but also the vast majority of patients had POAG in both the groups. The difference in treatment failure for tube shunts compared with TVT may have to do with different disease process in the valve group.

Connor et al^14^ carried out a similar investigation comparing the outcomes of trainee-performed glaucoma surgery. However, similar to the TVT study, trabeculec-tomy was compared with the nonvalved Baerveldt. A total of 153 patients were included and followed retrospectively over 1 year, and similar to this study, the authors found that although the preoperative IOP was higher in the tube shunt group, postoperative IOP was similar between groups.

The type and rate of surgery complications were a primary focus of this study. There were no intraoperative complications in the valve group, while four trabeculec-tomies were complicated by formation of buttonholes during conjunctiva closure. This difference is most likely secondary to the surgical technique. With valve surgery, the conjunctiva and Tenon’s capsule are removed as a unit to make a thicker covering for the valve. In a trabecu-lectomy, the conjunctival dissection off Tenon’s capsule to create a thin bleb is a more delicate technique, which may make buttonhole formation more likely. All of the buttonholes were repaired primarily and none of those eyes experienced leaks postoperatively. In another eye, the corner of the trabeculectomy flap was accidentally truncated; however, the surgery was completed successfully and the eye had no postoperative complications and an IOP of 9 mm Hg at 12 months.

Postoperative complications were stratified into three groups based on postoperative timeframe: Immediate (< 1 week), early (1 week to 3 months), and late complications (3-12 months). Eyes receiving an Ahmed valve seemed to have more immediate postoperative complications than trabeculectomy, mostly due to hyphema. In the valve group, hyphema occurred in a total of 12 (27.3%) patients and accounted for 55% of immediate postoperative complications. Prior studies have demonstrated hyphema occurring in 3 to 9% of valve implantation when performed by glaucoma specialists.^10,18,19^ One plausible explanation for the higher rate of hyphema is that more of the eyes receiving valve had neovascular glaucoma and therefore more new, friable vessels that were easily disrupted during surgery. Five eyes (8.5%) in the trab-eculectomy group had hyphema, which corresponds to published rates of 13 to 16%.^9,20^

Overfiltration can result in hypotony, defined as IOP less than episcleral venous pressure,^21^ which can result in serious complications, namely, choroidal effusions. For clinical purposes, eyes with IOP < 5 mm Hg are typically considered hypotonous, and overall, in this study, rates were low. Hypotony was more common in the trabecu-lectomy group, occurring in 6 (10.2%) eyes *vs* 2 (4.5%) in the valve group. Cases occurred equally early and late in trabeculectomy but only occurred after 1 week in the valve group. One eye with valve did develop choroidal detachment in setting of hypotony. The other hypoto-nous eye in the valve group had severe neovascular glaucoma, preoperative visual acuity of count fingers, and was pre-phthisic by 1 year. All the cases in the tra-beculectomy group were transient and improved by the next visit, except for two, which developed peripheral choroidal effusions. In prior studies, hypotony complicated approximately 32 to 45% of trabeculectomies by glaucoma specialists.^9,20^ When performed by residents, hypotony has been reported at a rate of 18 to 20% with clinical significance ranging from being a transient problem to requiring intervention and also returning to operating room for drainage of choroidals.^14,22^ This study is consistent in showing that residents tend to have lower rates of hypotony.

Wound leaks were rare in this study with only two instances in the trabeculectomy group and one in the valve group. The one in the valve group was only slightly Seidel positive and resolved with conservative measures. For trabeculectomy, one leak was treated with suture placement at the slit lamp. The other leak occurred in week 1 in an eye that was hypotonous immediately postoperative but was Seidel negative at that time. Studies have reported rate of wound leak at 9.7 and 0.7% for trab^9,20^ and valve^18^ respectively. No eyes developed endophthalmitis during the follow-up period.

Cataract development as a complication from surgery was uncommon but occurred more frequently in the trabeculectomy group: In five eyes (8.5%) *vs* two (4.5%) in the trabeculectomy and valve groups respectively. One eye in the valve group developed a cataract within the first week postoperatively and subsequently underwent cataract extraction. Overall, reoperation for any reason was necessary for 3.4% of trabeculectomy eyes and 9.1% of valves.

Following trabeculectomy, one eye developed a cho-roidal effusion and another developed suprachoroidal hemorrhage within a week postoperatively. Five eyes (11.4%) receiving Ahmed valve developed choroidal effusions from 1 week to 3 months postoperatively. The higher incidence of choroidal effusion in the valve group may relate to the larger average decrease in IOP since the preoperative IOP was higher in that group. With the published incidence choroidal effusion of 13 to 16 and 3 to 7% for valve^10,18^ and trab^9,20^ respectively when performed by glaucoma specialists, the rate of this serious complication is within acceptable range when performed by residents.

Mean visual acuity was significantly worse preop-eratively for eyes in the valve group. This difference persisted at 1 year; however, the VA in the valve group did not worsen more on average than trab over 1 year. This difference is likely explained by the predominance of neovascular glaucoma in the valve group, which portends worse visual outcomes. The C/D ratio was noted to be higher in the trab group preoperatively, which may be due to the fact that a larger portion of these patients had POAG and, therefore, likely a longer duration of disease. By 1 year, the C/D ratio was not statistically different between groups, indicating an increase in cupping in the valve group, which again reflects the larger portion of patients with more severe and acute secondary glaucoma. Each group experienced a statistically significant reduction in number of ocular antihypertensive medications by 1 year.

This study has several limitations. First, it is retrospective nature. Also, interpretation of the results of this investigation as a head-to-head evaluation of the Ahmed valve *vs* trabeculectomy is limited due to the different baseline characteristics of the eyes studied. With a larger portion of patients with diabetes and secondary glaucoma, specifically neovascular, in eyes receiving valve, the postoperative course may be altered based on underlying pathologic differences. Logically, one would expect more complications and possibly worse outcomes, which was not the case in this study. In addition, the demographic characteristics of the study group may be unique to the veteran population in this region and, therefore, may not be generalizable to other patient groups.

To date, this is the first known investigation of outcomes following Ahmed valve implantation *vs* trabeculec-tomy by residents-in-training. The results are encouraging that resident surgeons can safely perform these challenging surgeries and produce a satisfactory result with a good safety profile. Despite an influx of recent publications on resident-performed glaucoma procedures,^14,22-27^ a significant gap in the literature remains evaluating the plethora of procedures that residents perform on patients during their training. Not only will this research help monitor and shape surgical education, but it can also serve to provide evidence-based counseling for patients that supervised, resident-performed procedures are indeed safe and effective.

## CONCLUSION

Ahmed valve implantation and trabeculectomy produce significant reductions in IOP when performed by residents-in-training. Although complication profiles differ between procedures, both are safe and well tolerated when performed by resident physicians. This study provides support for evidence-based patient counseling that supervised, resident-performed Ahmed valve implantation and trab-eculectomy are indeed safe and effective.
